# Influence of Oxidation Level of Graphene Oxide on the Mechanical Performance and Photo-Oxidation Resistance of a Polyamide 6

**DOI:** 10.3390/polym11050857

**Published:** 2019-05-10

**Authors:** Roberto Scaffaro, Andrea Maio

**Affiliations:** Department of Engineering, University of Palermo, Viale delle Scienze, ed. 6, 90128 Palermo, Italy

**Keywords:** UV irradiation, reactive mixing, nanocomposites, durability, photo-stability, ATR/FTIR, photo-degradation, radical scavenging, antioxidant, UV-shielding

## Abstract

The aim of this work is to study the relationship between the chemical-physical properties of graphene oxide (GO) and the performance of a polyamide 6 (PA6) in terms of mechanical reinforcement and resistance to UV-exposure. For this purpose, two samples of GO possessing different oxidation degrees were added (0.75 wt.%) to PA6 by way of a two-step technique and the materials achieved were carefully analysed from a morphological, chemical-physical, mechanical point of view. Photo-oxidation tests were carried out to assess the performance of this class of nanohybrids after 240 h of UV-exposure. The results reveal that both nanocomposites exhibit enhanced mechanical performance and durability of PA6. However, the most oxidized GO led to a higher increase of mechanical properties and a stronger resistance to UV-exposure. All the analyses confirm that both GO samples are well dispersed and covalently attached to PA6. However, the higher the oxidation level of GO the stronger and the more extended the chemical interphase of the nanocomposite. As regards photochemical stability, both GO samples display UV-shielding capacity but the most oxidized GO also shows radical scavenging activity by virtue of its nanocavities and defects, imparted by prolonged oxidation, which endows PA6 with an outstanding durability even after 240 h of UV-exposure.

## 1. Introduction

Graphene oxide (GO), a representative pseudo-two-dimensional solid, is considered the main precursor of graphene. Achieved by oxidation of pristine graphite under acidic conditions, it displays an intriguing structure by virtue of a double honeycomb, constituted by both sp^2^ and sp^3^ hybridized carbon atoms and many highly reactive functional groups attached to the layers. Although it was synthesized by Brodie [1] more than one century ago, the structure of GO is still an object of discussion [2]. Although all the models agree on the presence of both sp^2^-conjugated atoms and oxygen-bearing end groups, the chemical nature of the oxygenated moieties is still uncertain [3]. Epoxy, hydroxyls, carboxylic acids, lactones, as well as four-membered ring ethers, ketones and quinones, are supposed to be placed either on the basal planes or at the edges [4,5]. The singular physical and chemical properties of GO stimulated the interest of materials scientists for the preparation of a wide range of—intercalated or exfoliated—polymer nanocomposites [6,7,8]. In this context, many studies reported on polyamide 6 (PA6)-GO nanocomposites [6,7,9,10]. Indeed, PA6 is an important engineered thermoplastic polymer with excellent mechanical properties and good processability but, conversely, its susceptibility to humidity, heat and UV exposure hinders its use in those application fields in which strong stability is strictly required [11,12].

While the effects of GO on the enhancement of mechanical, thermomechanical and electrical properties of PA6 have been abundantly investigated, the role of GO in the enhanced thermal oxidative stabilization of PA6 has only recently been reported [13,14] and no research has dealt with the effect of GO addition on the photo-stability of PA6. Indeed, other nanocarbons, such as graphene nanoplatelets and carbon nanotubes, were more recently tested as UV-stabilizers for polyethylene [15] and epoxy resins [16], demonstrating the possibility of enhancing the mechanical stability of the irradiated films. According to these studies, the antioxidant efficiency of such nanofillers seems to be correlated with the degree of their dispersion throughout the matrix. In the case of a hydrophilic polymer such as polyamide, amphiphilic GO may offer the possibility to ensure a higher dispersion degree with respect to graphene. GO has been shown to improve the UV-shielding of cellulose acetate-based films while retaining good transparency in the visible range [17]. However, the mechanical and spectroscopic durability of such materials has not been studied and the relationship between the physicochemical properties of GO and its UV-shielding capacity is far from being fully elucidated [17]. Even if its aromaticity is obviously lower than that of graphene and carbon nanotubes, the presence of quinone-like and polyphenol-like moieties within its honeycomb, as well as the large number of defect sites, may result in a strong antioxidant activity.

The aim of this work is to elucidate the effect of the physicochemical features of GO on its UV-stabilizing activity onto PA6. For this purpose, small amounts (0.75 wt.%) of two types of GO, characterized by different oxidation levels, were integrated into a PA6 matrix with a two-step technique and the mechanical and chemical-physical durability of these nanocomposites was compared to that of the pure polymer by UV-irradiating the samples for up to 240 h.

In order to assess the propagation of photochemical reactions throughout the bulk, spectroscopic measurements were conducted on both the exposed and unexposed sides of the irradiated films and the results were compared with the mechanical decay of such materials.

## 2. Materials and Methods

### 2.1. Materials

In this work, two types of GO with different oxidation degrees were used. The sample named GO-1 was synthesized according to our previous work [9]; meanwhile the sample named GO-2 underwent prolonged oxidation under acidic conditions, as reported in Reference [18].

PA6, Radilon S35 100 NAT (density = 1.14 g cm^−3^, viscosity index (in H_2_SO_4_) = 2.05 ml g^−1^, radius of gyration of 8 nm, was supplied by Radicinova, Bergamo, Italy. *N*,*N*-Dimethylformamide (DMF), formic acid (HCOOH) (>99%, ACS grade), water and ethanol (EtOH) were purchased by Sigma Aldrich, Saint-Louis, MI, USA.

### 2.2. Methods

**Scheme 1:**
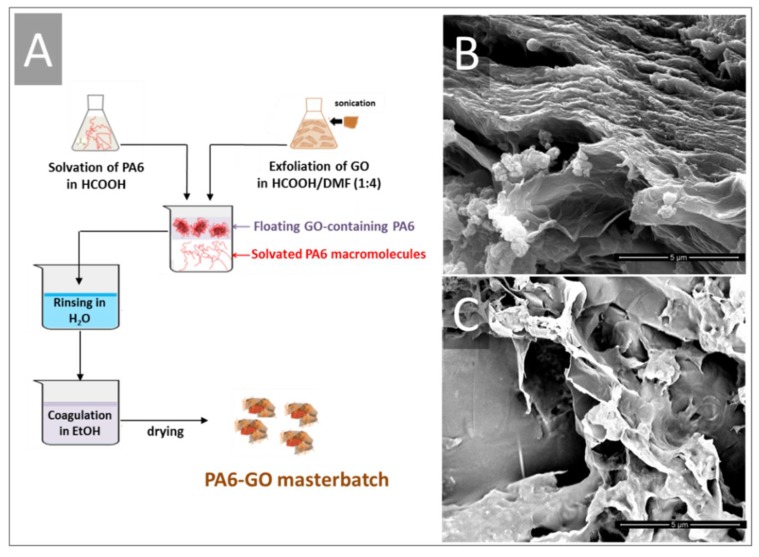
(**A**) Schematics of the preparation of the masterbatches and scanning electron microscopy (SEM) micrographs of the masterbatch containing Graphene oxide (GO)-1 (**B**) and GO-2 (**C**).

For the fabrication of PA6-based nanocomposites, a two-step process was adopted. As reported in our previous work [7], this technique involves the preparation of a masterbatch by wet phase inversion (step 1)—a technique commonly adopted to prepare porous structures [19]—and further melt processing with PA6 (step 2). For the generic preparation of a masterbatch (see Scheme 1), PA6 (5 g) was dissolved in HCOOH (50 mL) by heating (60 °C) under stirring, 0.5 g of GO (either GO-1 or GO-2) was dispersed into 200 mL of HCOOH/DMF (1:4), sonicated for 1 h. As GO slurry was added to the PA6/HCOOH solution, a phase separation instantly occurred with most of the PA6 remaining in solution, while brownish PA6-GO nanohybrids—which were found to float—were easily collected, rinsed in water, coagulated in ethanol, ground into fine powder (100–150 μm) and finally dried in a vacuum-oven at 90 °C overnight. The amount of dried masterbatch was found to be equal to 1.74 g and 2.19 g in the case of GO-1 and GO-2, respectively.

Finally, each sample was added to fresh PA6 until reaching a GO content of 0.75%, was pre-mixed at the solid state and fed to a Plasticorder 330 batch mixer (Brabender, Duisburg, Germany) for the further processing step. The parameters adopted, such as temperature (240 °C), rotor speed (64 rpm) and mixing time (8 min), as well as the choice of polymer: GO ratio used in the previous step, were set based on a previous study [7]. After being melt processed, the materials were collected within 2 min and quenched in liquid nitrogen. Thereafter, they were immediately ground into pellets, compression moulded and cut into specimens of appropriate geometry for further characterizations.

Spectroscopic analysis was performed on nanofillers, nanocomposites and extracted nanofillers. Spectroscopic measurements for each sample were conducted on 3 different zones for the sake of reliability.

Attenuated Total Reflection Fourier Transform Infrared (FT-IR/ATR) measurements were performed by means of a FT-IR/NIR Spectrum 400 spectrophotometer (Perkin-Elmer, Waltham, MA, USA) in the range 1800–1500 cm^−1^.

μ-Raman spectroscopy was performed in the wavenumber region of 1200–1900 cm^−1^ by a InVia instrument (Renishaw, Wotton-under-Edge, UK) (diode laser excitation = 633 nm, resolution = 1 cm^−1^).

UV-vis spectra were collected in the wavelength range of 190–1100 by using a Specord 252 spectrophotometer (Analytik Jena, Jena, Germany).

X-ray photoelectron spectroscopy (XPS) experiments were performed by using an ESCALAB MkII spectrometer (Thermo Fisher Scientific, Waltham, MA, USA) (pass energy = 20 eV) equipped with a standard Al Kα excitation source and a 5-channeltron detection system. 

X-ray diffraction (XRD) analysis was carried out by means of an Empyrean PANalytical II diffractometer (Malvern Panalytical, Worcestershire, UK), equipped with a Cu Kα radiation source (λ = 1.5406 Å). 

Scanning electron microscopy (SEM) imaging was carried out with an ESEM FEI QUANTA 200 microscope (Thermo Fisher Scientific, Waltham, MA, USA) on gold-sputtered samples. Transmission Electron Microscopy (TEM) analysis was carried out using a JEOL 2100 (JEOL, Akishima, Japan) operating at 200 kV.

Dispersion tests in distilled water were carried out to assess the capability of the two different samples of GO to remain in suspension (0.5 mg/L) and were corroborated by Atomic Force Microscopy (AFM) investigations (Multimode V Metrology scanning probe microscope) (Veeco, Plainview, TX, USA) and pH and zeta potential measurements (Zetasizer Nano ZS, Malvern Instrument Ltd., UK). 

Rheological analysis was performed in triplicate on disk-shaped specimens by using a parallel plate rheometer (Ares G2, *d* = 25 mm) in the frequency range: 0.1–500 rad/s at 240 °C and 10% strain amplitude.

Differential scanning calorimetry (DSC) was performed by a Perkin Elmer DSC7 (Perkin Elmer, Waltham, MA, USA) under N_2_ flow at a scanning rate equal to 10 °C/min. Crystallinity degree (χ) of PA6, was calculated according to Equation (1):

(1) $$\chi =\frac{\Delta H_{m}}{(1-\Phi_{w})\;\Delta H_{m}^{0}}\cdot 100$$

where $\Delta H_{m}$ is the melting enthalpy experimentally measured for each sample, whereas $\Delta H_{m}^{0}$ is that of a 100% crystalline PA6 (equal to 230 J g^−1^ [20]) and $\Phi_{w}$ is the filler weight fraction.

Tensile tests were performed using a dynamometer (Zwick-Roell Universal Testing Machine, Ulm, Germany), according to the ASTM D638–10 standard. The tests were performed on at least 8 replicates and the results were provided as mean values together with the corresponding standard deviations.

The films, 70 µm thick, were photo-oxidized in a Q-UV-Solar Eye weatherometer (Q-LAB, Westlake, OH, USA) equipped with eight UV-B lamps (λ = 313 nm). Each cycle comprised 8 h of light at *T* = 55 °C and 4 h of dark/condensation at *T* = 45 °C. The films were exposed for up to 240 h.

The eventual changes occurring in the specimens were evaluated by mechanical testing and ATR on both sides of films, that is, the exposed and unexposed sides, based on the consideration that, in the range of 1800–1700 cm^−1^, the penetration depth of the probe used does not exceed 35–40 µm. This novel approach provides quick information about the propagation of photo-oxidation paths in bulk, which is crucial for the retention (or decay) of mechanical properties in the materials.

## 3. Results and Discussion

### 3.1. Chemical-Physical Properties of GO-1 and GO-2

Undoubtedly, the extent of oxidation has strong repercussions on the chemical-physical features of GO. First, a different dispersibility in an aqueous medium was found. GO-1 tends to darken and compact, as a result of progressive dehydration and consequent re-stacking. The highly oxygenated GO-2 retains a light-yellow colour. 

**Figure 1:**
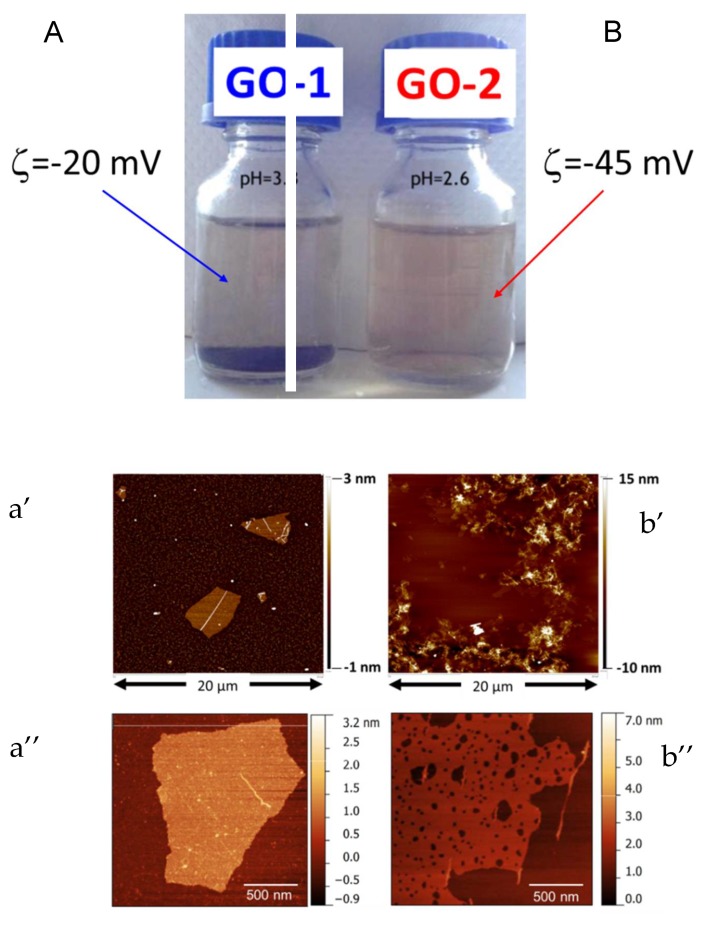
Digital photographs (with zeta potential values) and atomic force microscopy (AFM) micrographs of GO-1 (left, (**A**), (**a’**), (**a’’**)) and GO-2 (right, (**B**), (**b’**), (**b’’**)) suspensions in water at different magnifications.

**Figure 2:**
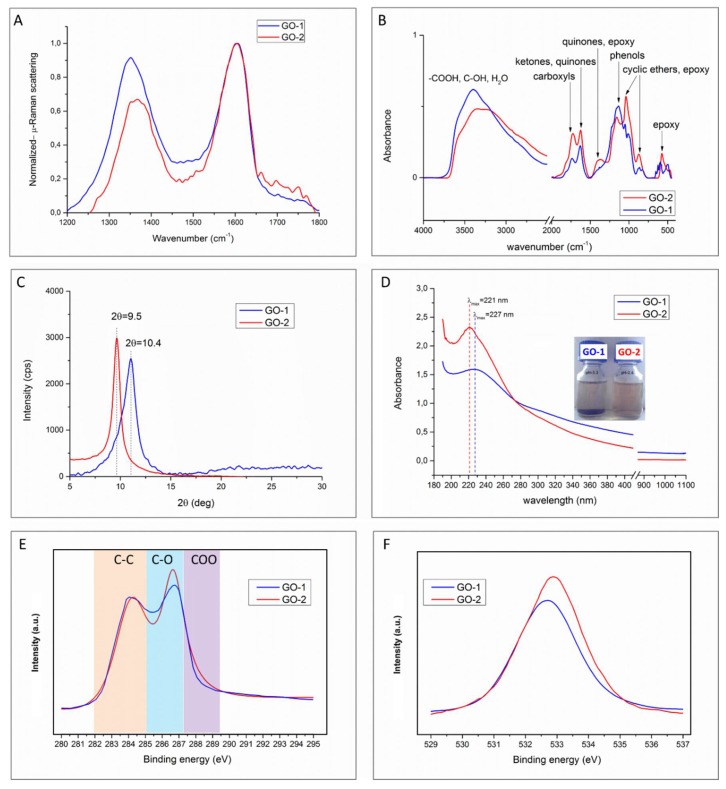
Physical and chemical characterization of GO-1 and GO-2: (**A**) μ-Raman spectra in the first-order phonon scattering; (**B**) Attenuated total reflection Fourier transform infrared (FTIR/ATR), (**C**) X-ray diffraction (XRD), (**D**) UV-vis, (**E**) X-ray photoelectron spectroscopy (XPS) C1s, (**F**) XPS O1s.

In Figure 1A,B, the dispersions (0.5 mg/l) of GO-1 and GO-2 in water after 10 days are reported, respectively. GO-1 and GO-2 showed dramatic differences from a chemical-physical point of view. The prolonged oxidation provided an impressive, enhanced stability in aqueous suspension over a wide period of time. Moreover, GO-1 (Figure 1A) required at least 1 h of ultrasonic treatment to achieve good dispersion in water, GO-2 (Figure 1B) remains in suspension if simply hand-shaken. Furthermore, the two GO samples displayed different acidities, with GO-2 dispersion resulting in a lower pH (2.6) than that of GO-1 (pH = 3.3), being equal to the concentration in water. Zeta potential measurements and AFM observations were performed to provide further insight into the status of lamellae. AFM observation of GO-1 dispersion, provided in Figure 1a’, revealed that GO-1 lamellae were more regular and smaller sized (*t* = 0.7 nm; *D* = 5 µm), owing to sonication, whereas GO-2 (Figure 1b’) displays extremely crumpled and wrinkled lamellae with a larger size distribution, ranging from hundreds of nanometres to several microns. Nevertheless, GO-2 has improved stability in aqueous media and a zeta potential (−45 mV) much more negative than GO-1 (−20 mV). The crumpling could be a direct consequence of the high density of epoxy and lactones moieties in the basal planes, developed within the re-arrangement of the structure during the aging, or due to the presence of oxidation debris. AFM analysis performed at a higher magnification of GO-1 (Figure 1a’’) and GO-2 (Figure 1b’’) enabled us to detect the presence of carbonaceous fragments (sub-micrometric sized) in both samples. If, in the case of GO-1, they might have been caused by high sonication times, for the simply hand-shaken GO-2 suspension one can hypothesize that the prolonged oxidation might have chopped up the basal planes of lamellae. As a result, GO-2 possesses an extremely wide lateral size distribution, ranging from 400 nm to 25–30 µm. Herein, we are not interested in explaining whether they are detrimental carbonaceous fragments or oxidative debris. Figure 2A reports μ-Raman plots of GO-1 and GO-2 in the range of 1200–1800 cm^−1^, that is, referred to the first order (one phonon) Raman region. The D peak (K point in the Brillouin zone) is located at 1350 cm^−1^, whereas the G mode (at point Γ) is usually centred at around 1600 cm^−1^. The D-band refers to the sp^3^ diamond-like carbons, whereas the G-band is ascribed to the sp^2^ hybridized carbons [21,22,23]. The Raman parameters allow the assessment of the oxidation degree and have an intensity ratio between D and G modes (*I*_D_/*I*_G_), which provides information about the size of sp^2^ domains and the band located at 1700–1780 cm^−1^, which is assigned to non-regular rings (5-7-7-5 Stone Wales defects, 5-8-5 rings resulting from a C di-vacancy, and so on [9,24,25,26]). By comparing the spectra of GO-1 and GO-2, normalized to G amplitude, strong changes were detected: *I*_D_/*I*_G_ ratio for GO-1 is shown to be higher than for GO-2, suggesting that the average size of sp^2^ domains dramatically increases in the order GO-1 < GO-2. Moreover, the increased intensity of the bands at around 1700–1780 cm^−1^ in GO-2 suggests a higher amount of non-regular rings [9,25]. Based on these results, it can be hypothesized that prolonged oxidation in GO-2 involved the amorphous carbon of GO, thus likely resulting in the formation of oxidative debris while preserving its aromaticity [27,28,29,30].

FTIR analysis of GO-1 and GO-2, reported in Figure 2B, confirmed that both samples have substantially the same functional groups but in different proportions. The bands related to carboxyl and epoxy moieties, located respectively at 1724 and 1025 cm^−1^, were more intense for GO-2. Beyond the little differences observed in the intensity of epoxy and carboxyl signals, they cannot explain the strong differences observed in macroscopic properties, such as stability of suspension and reactivity towards PA6. However, recent studies [31] put into evidence that the presence of debris interferes with the spectroscopic analysis of GO.

XRD plots are provided in Figure 2C. GO-1 and GO-2 displayed a broad band centred at 10.4° and 9.5°, respectively, while no diffraction peak at 26° (typical of pristine graphite) was detected. These results put into evidence that both samples underwent a full exfoliation during chemical oxidation and interlamellar spacing was found to be equal to 0.95 nm for GO-1 and 1 nm for GO-2. UV/vis spectra of the samples are provided in Figure 2D. Both samples display a maximum, which is in the 221–227 nm range, traditionally assigned to the π–π* plasmon peak at the transition of aromatic bonds, as previously reported for GO by several authors [4,32,33]. Furthermore, a shoulder, or at least a broad absorption region, can be detected in the wavelength range of 240–320 nm, likely arising from the overlapping of the modes associated to the absorption of epoxy groups (around 250 nm), to π–π* transition of aromatic C–C bond in partially restored C=C bonds (around 260 nm) [34], n–π* plasmon peak due to the transition of C=O bonds of the carbonyl groups (around 300 nm) [4]. This complex, extremely wide distribution is supposed to be due to the contribution of the different functional groups to the microstructural disorder, in agreement with FTIR and Raman spectroscopy results, and it is supposed to affect optical behaviour [34]. The degree of remaining conjugation can be determined by the λ_max_ of each UV/vis spectrum. It is observed that higher absorption, related to the π–π* transitions (conjugation), correlates with less energy needed for the electronic transition, which results in higher λ_max_. The lower λ_max_ exhibited by GO-2 suggests that, for an equal amount of each sample, the aromatic carbons of GO-2 are organized in graphenic islands bigger than those of GO-1, thus confirming the *I*_D_/*I*_G_ analysis outcome from Raman about the size of the aromatic domains. Furthermore, both GO samples are UV-absorbers, while in the visible range GO-2 display higher transparency than GO-1.

XPS results are provided in Figure 2E,F. C1s spectra of both samples (Figure 2E) display a peak centred at 284.5 eV that represents the graphitic carbon skeleton, a band centred at 287 eV, assigned to C–OH, –C–O–C– and a shoulder located at 289.4 eV attributable to –COO– bonds. The spectra, normalized to C–C signal, put into evidence that GO-2 possesses a higher oxygen content. A further confirmation of the higher oxygen content of GO-2 is provided by analysis of the O1s signal (Figure 2F), which was found to be stronger for the most oxidized GO sample. By comparing these findings with those carried out in FTIR, μ-Raman and UV-vis experiments, we can hypothesize that aging under acidic conditions leads to a considerably lower content of sp^2^ domains but larger in size [35].

### 3.2. Chemical-Physical Properties of PA6-GO Nanohybrids

**Scheme 2:**
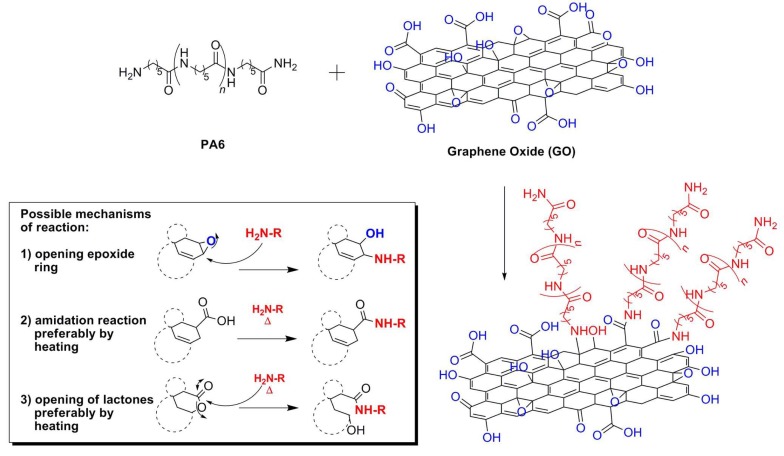
Schematics of possible reactions between polyamide 6 (PA6) and GO.

The great differences observed in the two batches of GO obviously affected both the structure and the properties of the PA6-based composites. During the wet phase inversion step, GO-2 showed higher affinity towards PA6, as it is able to drag a higher amount of PA6 into the aqueous phase during the coagulation. When GO-1(0.5 g) is used, the amount of dragged PA6 (1.24 g) is less than that (1.69 g) dragged by GO-2 (see again experimental part). At 60 °C, that is, during wet phase inversion, only epoxy moieties could react by opening the –C–NH– bond of the polymer or by directly reacting with –NH_2_ groups. Although it is quite difficult to measure the exact content of epoxy groups prone to react with PA6 moieties, we can suggest that the debris likely play a crucial role. They are supposed to act either as surfactants, thus stabilizing the macromolecules, and/or as nucleophilic agents, thus reacting with the amino and amide groups of the polymer, according to the schematics depicted in Scheme 2.

**Figure 3:**
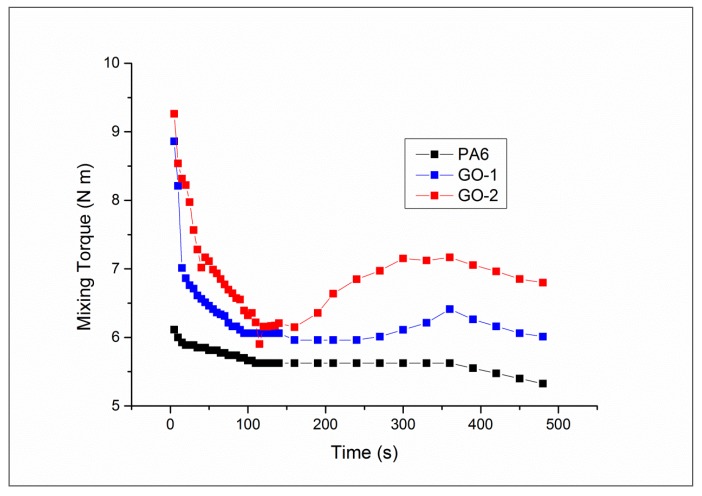
Mixing torque recorded for neat PA6 and nanocomposites containing GO-1 and GO-2.

Figure 3 presents the torque as a function of the mixing time of neat PA6 and its composites containing GO-1 and GO-2. As regards PA6, after an initial transitory in which the polymer decreases its viscosity, likely due to thermo-mechanical degradation, the torque achieves a plateau at about *t* = 100 s. When GO-1 is added, the torque, the initial value of which is higher than that of pure PA6 due to the presence of the rigid filler, decreases upon increasing mixing time, as observed in most polymer-filler systems investigated [36,37,38], until achieving a constant value of approximately 6 N·m at *t* = 150 s. At *t* = 240 s, torque starts increasing, achieving a maximum (6.3 N·m) at *t* = 360 s. When GO-2 is used, this behaviour appears more pronounced. In fact, after the typical transitory, at *t* = 150 s the torque starts increasing in a more remarkable way upon reaching a maximum (7 N·m) at *t* = 300 s. The final values of the torque were found to be equal to 5.4, 6.2 and 6.8 N·m for neat matrix, PA6/GO-1 and PA6/GO-2, respectively. The different behavior observed in these materials can be explained by considering the presence of phenomena with opposite repercussions on this property. In fact, thermomechanical degradative phenomena result in chain scissions pathways, which lead to a torque reduction. By contrast, eventual chemical reactions between filler and polymer, as well as filler exfoliation (although to a lesser extent), can lead to an increase of melt viscosity [13,14]. In fact, a maximum in mixing torque is typically observed in the case of reactive processing [13,14]. In the PA6/GO system, chemical reactions may involve –NH_2_ and –COOH end groups of polyamide and epoxy, lactones and carboxyl moieties abundantly present in GO, as schematized in Scheme 2. Beyond this, we cannot exclude the possibility that debris attach onto PA6 macromolecules, thus acting as bridge-molecules [31]. By virtue of its higher oxygen content, GO-2 has proven to be more reactive during the melt mixing. In fact, both onset and maximum in the torque plot occur earlier and to a greater extent if compared to the system containing GO-1. 

**Figure 4:**
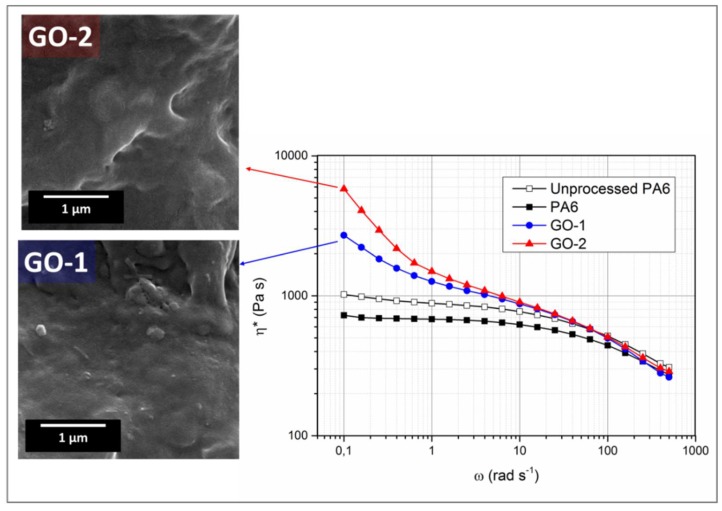
Rheology curves of unprocessed PA6 and of the samples prepared together with SEM micrographs of nanocomposites containing GO-1 and GO-2.

This assumption was corroborated by rheological analysis. The flow curves of nanocomposites prepared with GO-1 and GO-2 are provided in Figure 4, together with those of unprocessed and melt processed PA6. Both PA6 samples display Newtonian behaviour in the frequency range of 0.1–10 with a slight shear thinning at higher frequencies. However, melt-compounded PA6 shows a viscosity lower than unprocessed polymer, likely suggesting the occurrence of thermomechanical oxidative phenomena due to the long residence time in the mixer. By contrast, the two nanocomposite samples exhibited non-Newtonian behaviour within the whole frequency range investigated and viscosity values higher than those of neat polymer. Notably, the reactive melt mixing in the case of GO-2 results in more pronounced non-Newtonian behaviour and, if the yield stresses in the low frequency region in a nanocomposite system are usually governed by the nanofiller structured in a network [7,36], at higher frequencies (usually dominated by the polymer behaviour) the non-Newtonian behaviour persists and the complex viscosity of nanocomposites containing GO-2 was found to be higher than that of PA6-GO-1 system, also confirming the values of final torque discussed before. SEM micrographs of cryofractured samples of PA6/GO-1 and PA6/GO-2, provided in Figure 4, points out that a high level of matrix-filler adhesion was achieved in both cases, with lamellae totally embedded and surrounded by the polymer matrix. However, the differences observed in terms of rheological behaviour, among nanocomposites containing the same filler amount, suggests that interphase, in terms of interfacial adhesion, filler dispersion and chemical reactions is affected by the type of GO.

**Figure 5:**
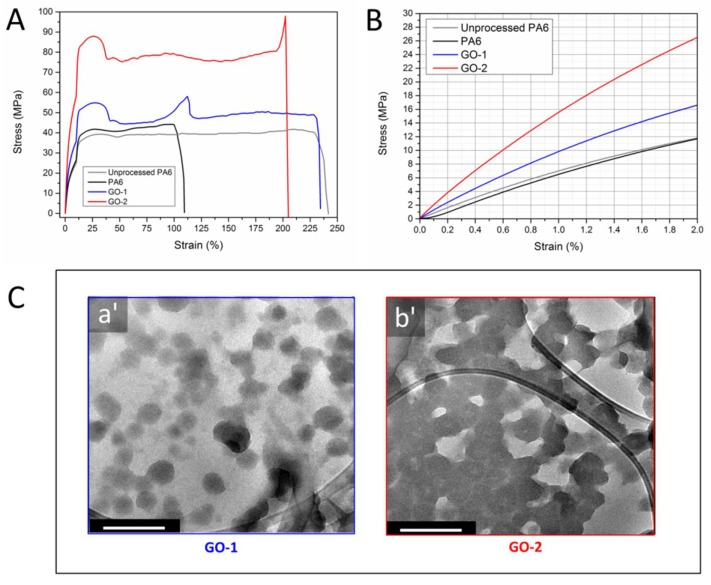
(**A**) Representative stress-strain curves of the samples prepared, (**B**) inset of low-strains region, (**C**) Transmission electron microscopy (TEM) micrographs of GO-1 (**a’**) and GO-2 (**b’**) containing nanocomposites (scale bar = 500 nm).

Figure 5A shows the representative stress-strain curves of nanocomposites containing GO-1 and GO-2 together with those of unprocessed and melt compounded PA6 for sake of comparison. Figure 5B provides a close-up of the low-strains region of each stress-strain curve. The main tensile properties, that is, elastic modulus (*E*), ultimate tensile stress (*TS*) and elongation at break (*EB*) of the samples investigated are reported in Table 1.

The analysis of stress-strain curves put into evidence that all the materials display ductile behaviour, characterized by a necking region after yield strain; thereafter, a plateau is observed for neat PA6 samples, whereas strain-hardening phenomena were detected for nanocomposites before failure. As regards neat PA6, melt processing was found to have negligible influence on *E* (from 638 to 668 MPa) and *TS* (from 40 to 42 MPa), while affecting *EB*, which proved to vary from 237% to 109%. This feature could likely be ascribed to thermo-mechanical degradation occurred during melt-compounding, due to the long residence time inside the batch mixer (8 min). Indeed, this result strongly agrees with the results of mixing torque and melt rheological analysis previously discussed. Adding GO-1 to PA6 determined an increase of stiffness and *TS* (~+40% relative increments), while preserving the stretchability of unprocessed PA6. The incorporation of GO-2 led to larger increments in terms of elastic modulus (+110%) and *TS* (+130%), whereas deformability slightly decreased (~−15%) with respect to unprocessed PA6, while being almost two-fold with respect to melt-processed PA6. First, the extremely high stretchability of nanocomposite films could be reasonably ascribed to the stabilising activity of carbonaceous fillers that prevent the thermo-oxidation of polymer during melt processing [39]. Moreover, the wrinkling of GO lamellae was found to be responsible for catalytic and antioxidant activity of these nanoparticles, since it enables them to absorb a wide variety of degradation products [40]. As regards the changes in terms of stiffness and tensile strength, it should be highlighted that elastic modulus is directly related to the volume of interphase, while *TS* is governed by interfacial strength [41,42,43]. Although both types of GO resulted in stiffer materials, the differences observed among nanocomposites possessing the same filler content suggest that the chemical-physical characteristics of the nanoparticles dramatically affect the interphase region, by influencing crucial features such as the extent of the interphase, the interfacial adhesion and the chain mobility of the polymer [7,44].

**Figure 6:**
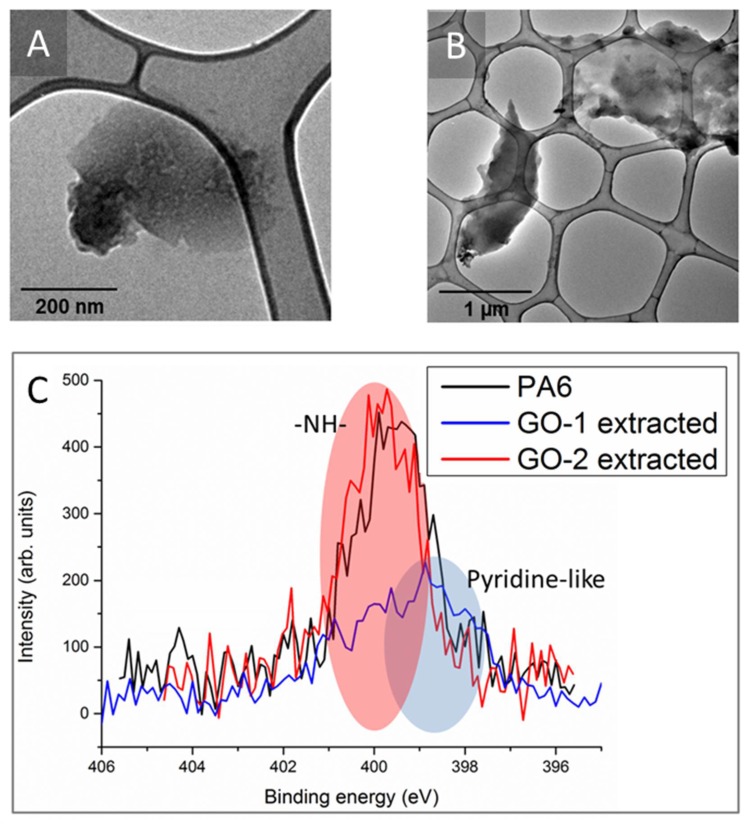
(**A**) TEM micrographs of GO-1 and (**B**) GO-2 extracted from nanocomposites; (**C**) N1s XPS of fillers extracted.

Panel C provides TEM micrographs of films containing GO-1 (C-a’) and GO-2 (C-b’). It can be seen that, in the case of GO-1 (lower aspect ratio, lower oxidation degree with respect to GO-2), a uniform dispersion was achieved with particle size ranging in the order of 200−300 nm. When GO-2 is used as a filler, it was observed the formation of a network, thus resulting in a huge increase of interphase volume. This feature can be explained by considering the much higher aspect ratio of GO-2 and the eventual reactions occurred between highly oxygenated lamellae and PA6 macromolecules, according to Scheme 2. A closer investigation of interphase was performed by coupling filler extraction with morpho-spectroscopic analysis, according to our previous work [6,45]. The nanocomposite samples were dissolved in HCOOH at *T* = 80 °C and repeatedly centrifuged aiming to extract free PA6 chains, whereas the insoluble fraction of each sample was collected and analysed. Figure 6 reports TEM micrographs of GO-1 (panel A) and GO-2 (panel B) extracted from their respective nanocomposites, together with N1s XPS spectra (panel C). Both samples proved to contain PA6, presumably reacted with graphene oxide, and this feature was easily monitored by N1s signal which, of course, are not present in graphene oxide. In particular, GO-1 lamellae extracted show polymer islands arranged into a pyridine-like structure [18], whereas GO-2 lamellae extracted display a uniform polymer-coating. In the latter case, the presence of thick polymeric layers covering GO lamellae is uniform, as already envisaged by the TEM analysis of nanocomposites (see again Figure 5C). Of course, the formation of a chemical interphase may have played a key-role in the extremely high *TS* increments observed in nanocomposite films containing GO-2, thus explaining the presence of strain hardening before failure and even the reduced ductility.

### 3.3. Photo-Oxidation Behaviour

**Figure 7:**
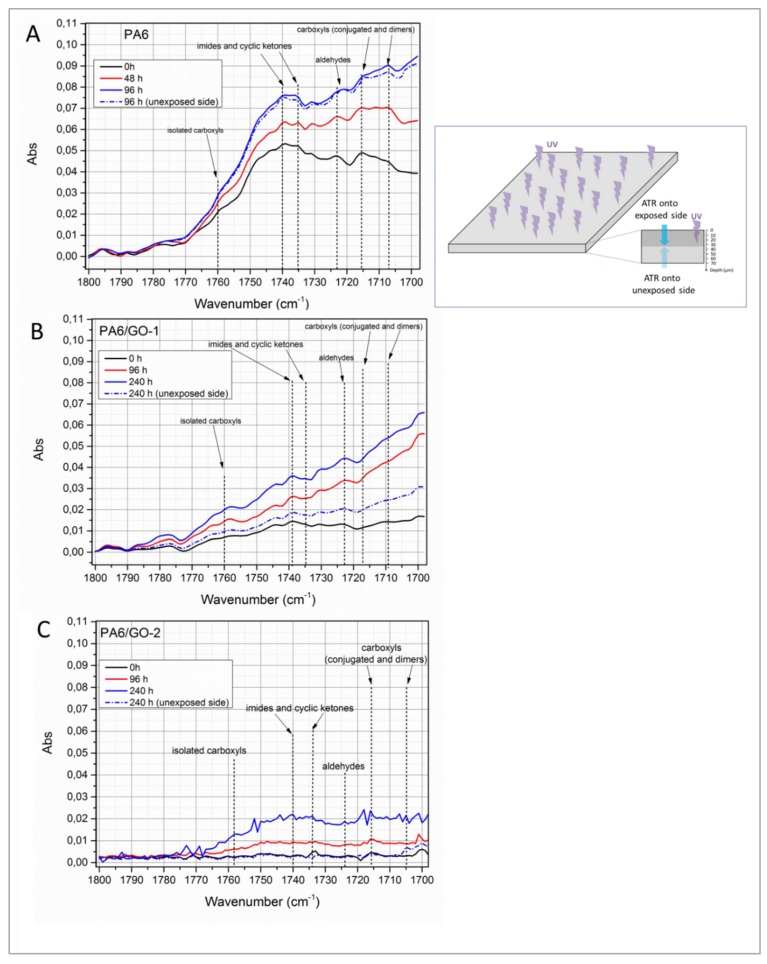
(**A**) FTIR/ATR spectra recorded in the carbonyl range 1800–1700 cm^−1^ of neat PA6; (**B**) nanocomposites containing GO-1 and (**C**) GO-2 together with a pictorial representation of ATR analysis performed to exposed and unexposed sides of the samples.

According to Cerruti et al. [46], it is possible to get information from FTIR, monitoring the build-up of carbonyl absorption within the range of 1700–1780 cm^−1^ due to primary and secondary photo-oxidation products. For this purpose, a detailed analysis of this spectral region was carried out and Figure 7A−C presents carbonyl domains at different irradiation times of the spectra recorded for PA6, PA6/GO-1 and PA6/GO-2, respectively.

Unirradiated PA6 (Figure 7A) presents a variegated spectrum, where it is possible to recognize a bunch of overlapped absorption bands. The presence of carboxylic acids, in dimeric, conjugated or isolated form, is confirmed by the bands centred at 1708 cm^−1^, 1715 cm^−1^ and 1760 cm^−1^, respectively [46]. Moreover, the modes centred at 1740 cm^−1^, 1735 cm^−1^ and 1723 cm^−1^ can be respectively ascribed to imides, cyclic ketones and aldehydes [46]. Upon increasing UV-exposure time, the same characteristic peaks can be detected in the spectra, but their intensities increase in a different manner. In fact, the kinetics of the formation and accumulation of photo-oxidation products are different each other, since—for instance—carboxylic acids (present in dimeric, conjugated or isolated form) are stable products, whose concentration grows owing to the oxidation of intermediate moieties, such as aldehydes and imines, and to the hydrolysis of amide groups [47]. Aldehydes can be already present in unexposed PA6 as a consequence of thermal oxidation during processing, as previously discussed, and can be then converted into carboxylic acids and α,β-unsaturated carbonyls [47]. On the other hand, imides and cyclic ketones are intermediates, less stable compounds, hence their presence could suggest that the reaction of photo-oxidation is ongoing [46,47]. Based on these considerations, one could measure the intensity ratio between the bands respectively centred at 1735 cm^−1^ (imides and ketones) and 1725 cm^−1^ (aldehydes). As clearly visible in Figure 7A, the intensity at 1735 cm^−1^ (*I*_1735_) is higher than that at 1725 cm^−1^ (*I*_1725_) in unirradiated PA6, whereas at 48 h and especially at 96 h of UV-exposure, *I*_1725_ becomes higher than *I*_1735_. Interestingly, PA6 film irradiated for 96 h shows practically the same spectrum on both sides, suggesting that photo-oxidation propagated in the bulk.

As regards nanocomposites containing GO-1 (Figure 7B), all the carbonyl species are scarcely present in unirradiated samples, likely due to the well-known antioxidant activity of nanocarbons [48,49,50,51], which could have protected the polymer from thermo-oxidative reactions during mixing, in fully agreement with the results of rheological and mechanical tests previously discussed. The build-up of carbonyl domain upon UV-exposure time in this case follows a different pathway, with the signals attributable to conjugated and dimeric carboxylic acids being almost indistinguishable each other but however more intense than those ascribed to imides and ketones. Notably, in unirradiated samples *I*_1735_ is close to *I*_1725_ value, whereas this latter becomes higher after 96 h and 240 h, thus suggesting that reaction is ongoing. Interestingly, after 240 h of exposure to UV radiation, the opposite side of the film displays a spectrum quite similar to that of unirradiated sample.

The nanocomposites containing GO-2 (Figure 7C) display a carbonyl domain deeply different from PA6 and PA6/GO-1. In fact, the chemical moieties typically detected in the other samples are scarcely present in this latter sample. The increment of intensities in the carbonyl region tends to zero even in the spectrum of films irradiated for 96 h and slightly increased after 240 h exposure, with values that—however—fall in the range of experimental noise. More interestingly, the opposite side of such films was found to be unaffected by UV exposure.

**Figure 8:**
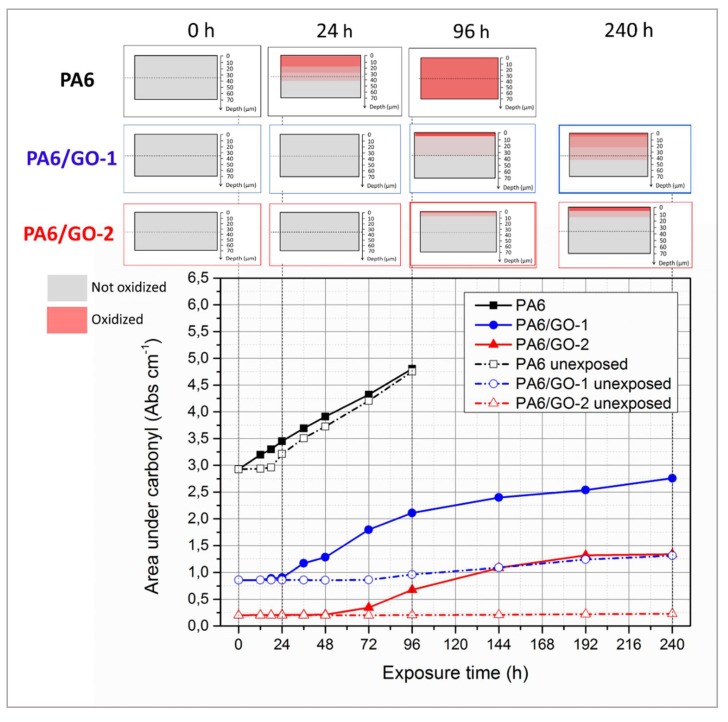
Integrated area under carbonyl performed onto exposed (solid line) and unexposed side (dash dotted line) for each sample, together with a pictorial representation of the propagation of photo-oxidative pathways.

The quantitative analysis of carbonyl build-up is provided in Figure 8, which reports the integrated areas under carbonyl, measured both onto exposed and unexposed side, plotted as a function of exposure time for all the samples, together with a pictorial representation of the propagation of photochemical reactions throughout the samples at certain time intervals. The first consideration concerns the initial values of this property, since unirradiated PA6 has an area of 3 Abs·cm^−1^, whereas those of PA6/GO-1 and PA6/GO-2 are respectively equal to 0.86 and 0.24 Abs·cm^−1^. This feature clearly shows once again that GO-1 and GO-2 prevented PA6 oxidation during melt processing. Furthermore, in PA6 this property is found to linearly increase upon irradiation time, and no induction time is detected in the exposed side of the films. In the unexposed side, a small induction time is observed, since area under carbonyl remains constant up to *t* = 24 h, thereafter the onset of carbonyl area occurs rapidly upon reaching the same value as the exposed side at *t* = 96 h. The onset of this property in the unexposed side suggests that photochemical reactions propagated in the bulk, whereas when the carbonyl area displays the same values in the exposed and unexposed face, it means that the reaction involves the entire sample (see pictorial representation of the propagation of photochemical reactions in Figure 8).

The system containing GO-1 displays a sigmoid curve in the exposed side, characterized by an induction time of 24 h, followed by a fast grow-up until reaching 2.2 Abs·cm^−1^ at *t* = 96 h. Thereafter, modest increments were observed, with this property being equal to 2.5 Abs·cm^−1^ after 240 h. The area measured in the unexposed side, instead, has proven to remain constant up to 72 h, thereafter it slight increased from 0.86 to 1.3 Abs·cm^−1^ after 240 h. This feature suggests that GO-1 nanofillers act as UV absorbers, resulting in UV shielding that, while being unable to arrest the propagation of photochemical reactions onto exposed surface, is capable of preserving the bulk. The area under carbonyls as a function of irradiation time in the exposed side of PA6/GO-2 films displays the same shape as that of PA6/GO-1 but in this latter case the induction time increased to 48 h, the grow-up proved to be slower and the final value was equal to 1 Abs·cm^−1^, i.e., practically the same as that of unexposed side of PA6/GO-1. More interestingly, in the unexposed side, no changes were detected within the whole time interval investigated. This behaviour is symptomatic of a multifunctional antioxidant activity. In fact, beyond the UV-shielding effect typical of carbonaceous nanofillers, GO-2 somehow hindered the propagation of photochemical reactions even throughout the exposed surface of films. A possible explanation of this multi-level protection may involve several factors: (i) GO-2 was found to absorb more than GO-1 in UV range (see again Figure 2C), thus resulting in a UV-shielding activity, (ii) GO-2 presents a larger amount of cavities, holes (Figure 1) and Stone-Wales defects (detected by Raman spectroscopy, Figure 2A) that are known to act as radical scavengers [49,50], (iii) also the extended chemical interphase may have prevented the propagation of degradation pathways, thus acting as a further barrier.

**Figure 9:**
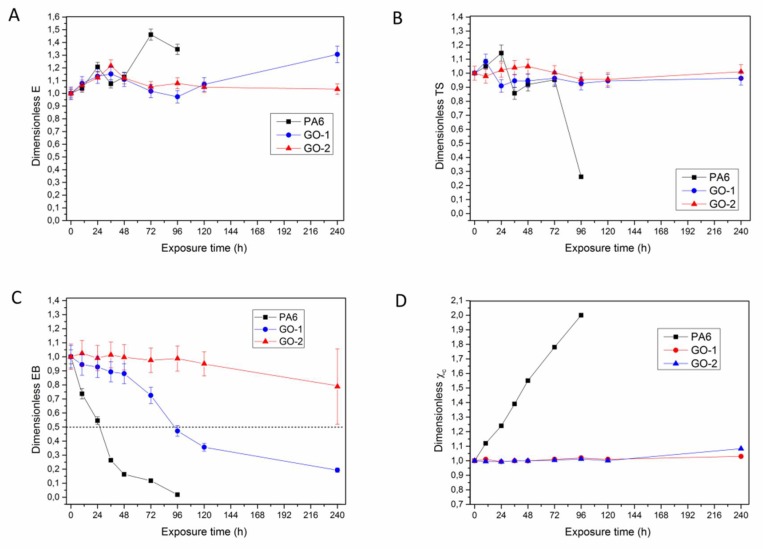
(**A**) Dimensionless *E*, (**B**) *TS*, (**C**) *EB*, (**D**) χ plotted as a function of exposure time for the samples investigated.

The evolution of mechanical properties, as well as eventual changes in crystallinity, during UV-exposure were monitored. Figure 9A–D respectively reports the dimensionless values of *E*, *TS*, *EB* and χ plotted as a function of the irradiation time. For all the samples, the dimensionless values of each property were calculated as the ratio between the value of the property at a given UV-exposure time interval and the value of that property at *t* = 0 h.

Elastic modulus (Figure 9A) was found to increase upon UV exposure time in the case of PA6, whereas no appreciable changes were detected for nanocomposites, regardless of the type of GO used. The increase of elastic modulus during photo-oxidation for a semi-crystalline polymer is attributable to the increase of crystallinity, due to the molecular weight reduction. The behaviour of dimensionless values of the crystallinity degree, χ, reported in Figure 9D, confirms this hypothesis, since this property proved to increase only in the case of PA6, while being substantially unaltered for nanocomposites containing either GO-1 or GO-2.

The same consideration can be made for dimensionless *TS*, Figure 9B. in fact, *TS* was found to increase for PA6 at low irradiation times, due to the higher crystallinity degree, thereafter this property was found to decay due to the increasing brittleness of the samples, which resulted in a premature failure. Nanocomposites display negligible changes during UV-exposure (±10–12% with respect to the initial value).

The analysis of dimensionless *EB*, instead, is a main concern, since this parameter is correlated with the oxidation level of a material. In fact, as the reaction propagates in the bulk, *EB* decays in such a rapid way [52].

As regards PA6 films, *EB* was found to decrease rapidly and monotonically with irradiation time. After 96 h UV-exposure, the excessive brittleness of the specimens made it impossible to continue the experiments. For nanocomposites containing GO-1, a slow decrease was detected until 96 h, thereafter dimensionless *EB* reduced to 0.2. However, the samples irradiated at 240 h retained a ductile behaviour. The nanocomposites containing GO-2 showed impressive stretchability retention, since dimensionless *EB* was found to remain close to 1 within the time investigated, with a slight reduction (0.8) observed only after 240 h of UV-exposure. Even in this case, a ductile mode in the failure was observed in all the irradiated samples, although the data scattering at this exposure level was found to increase. Furthermore, it was determined the half life time (HLT), i.e., the time necessary to achieve the halving of initial *EB* value [48]. This is a technological parameter to quantitatively assess the effect of the type of filler on the photo-oxidation kinetics. HLT proved to be equal to 26 h for PA6, while being enhanced to 96 h in the case of PA6/GO-1 nanocomposite. Notably, for the materials containing GO-2 no HLT was observed within the time interval investigated, even if some specimens experienced HLF at 240 h (see error bars in Figure 9D). The data point out that there is a correlation between HLT and ATR analysis of unexposed side of the films. In fact, it can be observed that HLT occurs when carbonyl band area of the unexposed side of the films begins to grow, with this latter feature indicating that photochemical reactions propagated into the bulk.

Although the energy of solar radiation would not be sufficient to cause the direct homolytic scission of C–N bonds, the light absorption can promote homolytic scission of the carbon-hydrogen bonds, owing to the presence of impurities such as catalyst residues, defects in the specimens, and carbonyl or peroxide species formed during melt processing [47,53]. As reported in scientific literature [48,54], carbonaceous fillers may act as UV absorbers, as chain breaking donor/acceptor of free radical species, as quencher of excited states such as active carbonyl species, and as hydroperoxides decomposer. The oxidation rate in PA6 is mainly determined by radical-forming reactions during UV-exposure, owing to the direct photolysis of the amide bonds [53]. These hypotheses clearly explain why GO-based nanocomposites showed an improved photo-stability compared to neat PA6. Indeed, due to the presence of aromatic species, defects and cavities within its honeycomb, GO-2 could act as radical scavenger and the formation of an extended chemical interphase in the materials loaded with GO-2 resulted in a sort of impermeability towards the propagation of oxidation reactions, as confirmed by ATR performed on the opposite sides of the samples. The concomitance of all these factors could explain the excellently enhanced photo-stability.

## 4. Conclusions

In summary, the effect of the oxidation level of GO on the mechanical and photo-resistance performance of PA6 was carefully analysed. A simple two-step strategy was implemented, enabling the full exploitation of the reactivity of GO to prepare PA6-based nanohybrids with outstanding chemical-physical properties by integrating into the polymer matrix two GO samples (0.75 wt.%) characterized by different oxidation degrees. The results confirmed that excellent levels of dispersion and interfacial adhesion are achieved by virtue of chemical reactions between host polymer and nanofillers. Consequently, mechanical performance of nanocomposites dramatically enhanced. Adding 0.75% GO-1 led to materials with +40% stiffness and *TS* relative increments, whereas GO-2 resulted in even higher increments (up to +130% in strengthening), while substantially preserving the stretchability of unprocessed PA6. Similarly, the resistance towards UV exposure dramatically enhanced and a correlation between oxidation level of GO and photostability of nanocomposites was found. In fact, HLT of the neat polymer is equal to 24 h, while being enhanced to 96 h and to more than 240 h by incorporating GO-1 and GO-2, respectively. The oxidation rate in PA6 is mainly governed by radical-forming reactions during UV-exposure, owing to the direct photolysis of the amide bonds. Indeed, due to the presence of aromatic species, both GO samples were able to increase photo-oxidation resistance of PA6 via a UV-shielding mechanism, by preventing or at least delaying the propagation of photochemical reactions. Owing to the presence of defects and cavities within its honeycomb, GO-2 could act as a radical scavenger and the formation of an extended chemical interphase in the materials containing GO-2 resulted in a sort of further impermeability towards the propagation of oxidation reactions, as confirmed by ATR performed onto the opposite sides of the samples. The concomitance of all these factors can reasonably explain the excellently improved photo-stability. Nanocomposites containing 0.75 wt.% GO-2 have proven to block the penetration of photo-oxidation pathways, whose depth was found to be less than 30–35 μm. These results suggest that a thin film of PA6 containing 0.75% GO-2 may be able to block UV-radiations, thus finding application as external coating for multi-layer polymeric films. In this regard, future developments of the present study could involve the preparation of a bilayer constituted by a UV-resistant coating to assess the possibility of preserving UV-sensitive polymers, such as neat PA6 itself but even other resins particularly susceptible to photo-degradation, thus extending the class of polymers that can be employed for outdoor applications, including components for photovoltaic cells, automotive, as well as materials for packaging or for the preservation of cultural items.

## Figures and Tables

**Table 1:** Tensile properties of the samples investigated expressed as mean values ±SD.

Sample	*E* (MPa)	*TS* (MPa)	*EB* (%)
PA6 (unprocessed)	638 (±49)	40 (±0.8)	237 (±12)
PA6	668 (±38)	42 (±1.0)	109 (±14)
GO-1	924 (±41)	55 (±3.6)	233 (±20)
GO-2	1407 (±52)	92 (±7.2)	207 (±16)
